# The Role of the Gut Microbiota and the Immune System in the Development of Autism

**DOI:** 10.7759/cureus.11226

**Published:** 2020-10-28

**Authors:** Rayan M Lungba, Seyad Zulficar Ali Khan, Uvie Ajibawo-Aganbi, Maria V Perez Bastidas, Swathi Veliginti, Sania Saleem, Ivan Cancarevic

**Affiliations:** 1 Medicine, California Institute of Behavioral Neurosciences & Psychology, Fairfield, USA; 2 Internal Medicine, California Institute of Behavioral Neurosciences & Psychology, Fairfield, USA; 3 Family Medicine, Ministry of Health Oman, Salalah, OMN; 4 Faculty of Health Sciences, California Institute of Behavioral Neurosciences & Psychology, Fairfield, USA; 5 Research and Development, California Institute of Behavioral Neurosciences & Psychology, Fairfield, USA

**Keywords:** autism, autism spectrum disorders, gut microbiota, immune system, gut flora

## Abstract

Autism‌ ‌spectrum‌ ‌disorders‌ ‌(ASDs)‌ ‌are‌ ‌neurodevelopmental‌ ‌disorders‌ ‌that‌ ‌present‌ ‌with‌ ‌social‌ skills‌ ‌and‌ ‌communication‌ ‌challenges,‌ ‌restricted‌ ‌interest,‌ ‌and‌ ‌repetitive‌ ‌behavior.‌ ‌The‌ ‌specific‌ cause ‌of‌ ‌autism‌ ‌is‌ ‌not‌ ‌well‌ ‌understood‌ ‌yet.‌ ‌However,‌ ‌numerous‌ ‌studies‌ ‌indicated‌ ‌that‌ ‌environmental‌ ‌and‌ ‌genetic‌ ‌factors,‌ ‌dysregulated‌ ‌immune‌ ‌response,‌ ‌and‌ ‌alterations‌ ‌to‌ ‌the‌ ‌balance‌ ‌and‌ ‌content‌ ‌of‌ ‌the‌ ‌gut‌ ‌microbiota‌ ‌are‌ ‌implemented‌ ‌in‌ ‌the‌ ‌development‌ ‌of‌ ‌autism.‌ ‌Many‌ ‌non-pharmacological‌ ‌interventions‌ ‌are‌ ‌nominated‌ ‌to‌ ‌manage ‌autism,‌ ‌including‌ ‌family‌ ‌support‌ ‌services‌ ‌and‌ ‌psychoeducational‌ ‌methods‌. Moreover,‌ ‌different‌ ‌pharmacological‌ ‌therapy‌ ‌modalities‌ ‌are‌ ‌recommended‌ ‌for‌ ‌children‌ ‌with‌ ‌ASD.‌ ‌Learning‌ ‌more‌ ‌about‌ ‌the‌ ‌brain,‌ ‌immune‌ ‌system, ‌and‌ ‌gut‌ ‌connections‌ ‌could‌ ‌assist‌ ‌in early‌ ‌diagnosis‌ ‌and‌ ‌treatment‌ ‌of‌ ‌this‌ ‌devastating‌ ‌neurodevelopmental‌ ‌disorders‌ ‌as‌ ‌an‌ ‌early‌ ‌intervention‌ ‌in‌ ‌ASD‌ ‌could‌ ‌improve‌ ‌a‌ ‌child's‌ ‌overall‌ ‌development.‌ We‌ ‌gathered‌ ‌data‌ ‌from‌ ‌relevant‌ ‌previously‌ ‌published‌ ‌articles‌ ‌on‌ ‌PubMed‌ ‌to‌ ‌evaluate ‌the‌ ‌role‌ ‌of‌ ‌the‌ ‌gut‌ ‌microbiota‌ ‌and‌ ‌the‌ ‌immune‌ ‌system‌ ‌on‌ ‌the‌ ‌development‌ ‌of‌ ‌autism.‌

## Introduction and background

Compared to other body areas, the gut has the most significant number of microorganisms, more than a trillion, with various species [1]. Gut Microbiota referred to microorganisms that live in our digestive tract; it is crucial for our health [2]. Altered gut microbiome composition called Dysbiosis is associated with many inflammatory diseases, obesity, auto-immune diseases, and cancers [3-5]. There are numerous communication paths between the gut and the brain through the short-chain fatty acids, immune system, and vagus nerve [6]. Any imbalance in gut microbiota can affect these paths, modulate brain functions, and result in many neuropsychiatric disorders [6]. Understanding the molecular and cellular modifications co-occurring with Dysbiosis might help developing new treatment approaches [5]. The priority could be re-establishing gut microorganisms’ diversity by introducing certain microbes, fecal transplant therapy, and using specific drugs, antibiotics, or probiotics [5].

Autism spectrum disorders (ASDs) refer to a wide range of disorders present with social skills and communication challenges, restricted interest, and repetitive behavior [7,8]. The rate of autism has been dramatically increasing worldwide and also in the United States [9,10]. As reported by the Center for Disease Control and Prevention (CDC), autism affects one out of 59 children in the United States, with boys four times more likely to be impacted than girls [11]. Autism signs can develop slowly and become apparent in early childhood [9]. Brain changes in autism are not restricted to a single brain region; different neuroimaging techniques detected structural abnormalities in various brain areas of autistic children such as the limbic system, corpus callosum, temporal and frontal cortex, cerebellum, and basal ganglia [12]. Signs and symptoms of autism may include but are not limited to repeating specific behavior, difficulty adapting when a routine change, and avoiding eye contact. 

The precise etiology of autism is not yet known [13,14]. However, evidence indicated that genetics and environmental factors, abnormal immune response, and altered gut microbiota composition are linked to the development of autism [15-17]. Regarding genetic elements, it is speculated that some genetic mutations arise in the majority of children with autism [18]. Notably, not all genetic modifications follow the same pattern; they encompass numerous types of mutations [18]. Of these mutations, autism has been linked to a transformation of methyl binding protein 2 (mecp2) and genetic polymorphism of cytochrome P450 enzymes (CYP450), specifically, CYP27B1, which is necessary for vitamin D metabolism [19]. Recent studies in the brain of both autistic children and their mothers found autoantibodies targeting brain proteins [20]. Data suggest that there may be a defect in signaling paths shared by the central nervous system and the immune system in children with autism and unbalanced levels of inflammatory cytokines and inflammation-related transcription factor nuclear factor kappa-B (NF-kB) [21-23].

Many non-pharmacological interventions are nominated to manage autism, including family support services and psychoeducational methods [24,25]. Different pharmacological treatment modalities are recommended for children with autism [26]. Pharmacological therapy includes antipsychotics, central nervous system stimulant drugs, and selective serotonin reuptake inhibitors (SSRIs) [26]. Of these agents, antipsychotics are the most extensively studied for controlling autism symptoms [26]. When administered in a low dose, antipsychotics have been found to decrease social withdrawal and stereotypes repetitive behavior, temper tantrums, and self-abusive behavior [26].

Learning more about the brain, immune system, and gut connections could assist early diagnosis and treatment of this devastating neurodevelopmental disease as an early intervention in autism could improve a child's overall development. This literature review article aims to evaluate the neurobiology of autism and the role of the gut microbiota and immune system response in developing autism in children. We searched Pubmed as the primary source to gather the related studies. We selected articles based on details and relevancy and included randomized clinical trials, animal studies, systematic reviews, and observational studies.

## Review

The gut microbiota and autism 

Research indicates that there are discrepancies in the composition of the gut flora between autistic children and controls, with an excess quantity of members of Clostridium and Sutterella genus found in the gut of children with autism [27]. Further evidence from animal models illustrated that specific microbial shifts in the digestive tract may result in changes similar to the clinical picture of autism, with suggested mechanisms involving immunological and metabolic aberrations, abnormality in fermentation processes/products toxin production [27].

Finegold et al. examined stool specimens from 33 children with autism aged two to nine years with gastrointestinal (GI) abnormalities and 13 control children with no autism or GI disorders to assess the amount of Clostridium perfringens (C. perfringens) and C. perfringens toxin genes in their gut [27]. They conducted a quantitative comparison of all Clostridium species and C. perfringens strains from the fecal microbiota, also separated C. perfringens pieces, and performed a polymerase chain reaction (PCR) analysis for the primary C. perfringens toxin genes, like C. perfringens enterotoxin gene, iota, epsilon, alpha, beta, and beta2 [28]. They found that children with autism and GI disease harbor statistically significant greater counts of C.perfringens in their gut compared to the control subjects (p = 0.031) [28]. Furthermore, the incidence of beta2 toxin-producing C.perfringens was considerably greater in children with autism than control subjects (p = 0.014) [28]. Also, children with autism have statistically significant larger counts of the beta2 toxin gene in their GI tract (p = 0.015) [28]. Researchers did not recognize other toxin genes, like epsilon, iota, and beta toxin genes from either autism subjects or control children [28]. Simultaneously, the investigators found the alpha-toxin gene in all studied C.perfringens strains, and they distinguished the C.perfringens enterotoxin gene only in three autistic and one control subjects [28].

Tomova et al. investigated fecal microbiota changes and the frequently existing digestive tract disorders in children with autism [29]. They examined fecal microflora of ten children with autism, nine siblings, and ten healthy control children by real-time PCR [29]. The autistic children fecal microbiota expressed a considerable reduction of the Bacteroidetes/Firmicutes ratio and an increase in Lactobacillus species [29]. Moreover, it indicated an increase in the incidence of Desulfovibrio species in autistic children and a critical positive association between the severity of the gastrointestinal tract dysfunction and the severity of autism [29].

Investigations showed a microbial shift in the digestive tract of children with autism, with a significant increase in the number of Desulfovibrio species, Lactobacillus species, and C. perfringens. Likewise, animals with similar microbial changes presented with a clinical picture of autism. Concluding, the gut microbiota is necessary to maintain the digestive system's physiological state; alteration of this intestinal flora has been associated with autism.

The influence of TNF-α, IL-8, and IL-6 on autism

Autism is associated with central nervous system inflammation, which leads to an imbalance in the plasma level of some inflammatory markers [13]. Identifying and measuring autism-specific inflammatory biomarker levels may facilitate the rapid diagnosis and designation of new therapeutic targets [20]. Of these biomarkers, autism has a link to tumor necrosis factor-alpha (TNF-α), interleukin 8 (IL-8), interleukin 6 (IL-6) [30-32].

Alzghoul et al. conducted a study in Jordanian children to measure and compare TNF-α, IL-8, and IL-6 in autistic children, their unaffected siblings, and unrelated healthy controls [11]. After investigations, they reported an exclusive elevation in the plasma levels of TNF-α and IL-8 in autistic children in contrast to their siblings and unaffected healthy control group (p < 0.001, p < 0.001) [11]. With no significant difference in the plasma levels of the formerly mentioned cytokines in the siblings and unrelated control subjects [11]. Regarding IL-6, they demonstrated an increase in its plasma levels in both autistic children and their siblings, compared to the unrelated healthy control group (p < 0.05) [11]. 

Wei et al. had immunohistochemistry studies that indicated the association of IL-6 with autism [23]. They employed in vitro adenoviral gene delivery technique to overexpress IL-6 in cultured cerebellar granule cells [23]. Aimed to examine how IL-6 affects neural cell development and function [8]. They proposed that the high levels of IL-6 in the brain of an autistic child could modify neural cell adhesion, migration, and result in an imbalance of inhibitory and excitatory circuits [23]. They revealed an increase in the plasma levels of TNF-α, IL-8, and IL-6 might be partially responsible for developing autism [23].

Several studies measured and compared the plasma level of TNF-α, IL-8, and IL-6 in autistic and control subjects. Results indicated a statistically significant increase in the level of TNF-α, IL-8 (p < 0.001), and IL-6 (p < 0.05). The influence of these inflammatory cytokines on autism is proposed.

Other inflammatory factors associated with autism

Studies indicated that brain inflammation has a significant role in the pathophysiology of autism. Theoharis C Theoharides et al. reported that mast cells (MCs) are involved in the development of autism, which worsens with stress [33]. Stimulation of mast cells by neuropeptides like the corticotropin-releasing factor (CRF) and neurotensin (NT) can lead to the release of inflammatory and neurotoxic mediators [33]. These mediators disrupt the blood-brain barrier, stimulate microglia, and cause focal inflammation [33]. This hypothesis was reinforced by detecting a high amount of MCs, CRF, and NT in the brain of children with autism in contrast to control subjects [33]. Clinical trials confirmed that and noted a statistically significant improvement of autism symptoms by using anti-inflammatory and antioxidant agents [33].

Autism is also related to dysregulation of some other inflammatory factors [21]. In 2018 a study assessed the messenger ribosomal nucleic acid (mRNA) expression levels of transforming growth factor -B (TGF-B), chemokine ligand 8 (CXCL8), interleukin 1B (IL-1B), interleukin 2 (IL-2), interleukin 4 (IL-4), and interleukin 17 (IL-17) in whole blood samples of 30 autistic children and 41 age and sex-matched healthy children with means of real-time PCR [21]. Results showed that IL-17 is considerably stimulated in autism subjects (P < 0.0001) when compared to healthy children [21]. IL-2 was reasonably negatively regulated in all autistic children (p < 0.0001) [21]. No marked discrepancy has been verified in other inflammatory cytokines’ expression levels between autistic and control subjects [21].

Interleukin-4 (IL-4) and interleukin-10 (IL-10) are recognized mostly as anti-inflammatory cytokines [34]. However, some inflammatory-related disorders in newborns have been attributed to high IL-4 and IL-10 concentrations [34]. Alan Leviton et al. supported this hypothesis [34]. They found fewer scores on components of a series of neuro-psychological tests-II, oral, written language scales-II, and Wechsler individual achievement-III test assessments among children who possessed top quartile concentrations IL-4 or IL-10 on day 21 or 28 after delivery [34].

Ahmad et al., in their clinical trial, assessed the effect of T cell immunoglobulin and mucin domain-3 (TIM-3) signaling in the development of autism [35]. They reported that children with autism considerably generated TIM-3, CD11a,b, CD14, chemokine receptor type 5 (CXCR5), interleukin 1B (IL-1B), and interferon-gamma (IFN-γ) related mRNA, and protein expression levels in contrast to control children [35]. Further, instructed that TIM-3 signaling detection could facilitate the early diagnosis of autism [35].

There is an intimate and complicated relationship between the brain and the immune system; the pathophysiological changes in autism have been correlated to certain inflammatory factors’ elevation. Understanding each element’s role is critical for early testing and diagnosis of autism and could be studied to develop future interventions.

## Conclusions

Several studies showed a significant variation in the gut microbiota in children with autism compared to control children with a remarkable amount of Desulfovibrio species, lactobacillus species, and C. perfringens. Research also reported that some inflammatory cytokines’ plasma levels were higher in autistic children with a notable amount of tumor necrosis factor-alpha (TNF-α), interleukin 8 (IL-8), interleukin 6 (IL-6). Further, there is an elevation of some anti-inflammatory cytokines like Interleukin-4 (IL-4) and interleukin-10 (IL-10) in children with autism. These could be targeted areas to manage autism and provide better developmental outcomes. However, it is not clear yet if the gut microbiota treatment and the blunt of the inflammatory cytokine signals courses could treat autism. More research is needed to elaborate on that.
